# Redefining ‘stress resistance genes’, and why it matters

**DOI:** 10.1093/jxb/erw323

**Published:** 2016-10-04

**Authors:** Lyza G. Maron, Miguel A. Piñeros, Leon V. Kochian, Susan R. McCouch

**Affiliations:** ^1^School of Integrative Plant Science, Plant Breeding and Genetics section, Cornell University, Ithaca, NY 14853, USA; ^2^Robert W. Holley Center for Agriculture and Health, USDA-ARS, Cornell University, Ithaca, NY 14853, USA; ^3^Current address: Global Institute for Food Security, University of Saskatchewan, Saskatoon, S7N 5A8, Canada

**Keywords:** Aluminum, gene, gene expression, ontology, resistance, stress, stress resistance gene, tolerance.

**Many plant biologists work on the identification of genes related to abiotic stress resistance, but the term ‘stress resistance gene’ is widely used without proper definition. Here it is argued that there is a need to update our understanding of this term and for standardization to facilitate integration of research data.**

The term ‘stress resistance gene’ [replace ‘stress’ by heat, drought, salinity, aluminum (Al), and so on] is widespread in the literature, without consensus about its meaning. Given what we have learned from over two decades of gene function studies, we suggest that there is a need to revisit how we assign gene function, and judiciously adopt standardized protocols and the use of controlled vocabulary. Blum (2016) expressed the need for a proper definition of ‘stress’, not solely for scientific clarity, but as a prerequisite for the proper design of experiments and interpretation of research outcomes. Similarly, the way we define and assign gene function has critical implications for how we approach gene discovery and how we apply this knowledge in breeding or biotechnology.

## Plant responses to stresses can be complex and overlapping

Abiotic conditions vary over time, and diverse abiotic stresses often occur in combination and/or consecutively within a plant’s environment. A plant’s response to stress is multifaceted, varying in time (i.e. early versus delayed responses) and space (i.e. whole plant, organs, tissues, or cell types), and is controlled by complex networks that can include multiple signaling pathways. From an evolutionary perspective, it is incongruous to think that plants would have independently evolved unique resistance mechanisms (and correspondingly unique ‘resistance genes’) in response to each stress. Nevertheless, the literature is rich with examples of genes characterized under a single form of stress. In addition, plant responses to stress are often studied in highly controlled environments, where the effect of a particular stress can be studied in isolation. While these approaches have contributed significantly to our understanding, they only yield limited conclusions about how plants respond to the complexity of stress signals in the environment. Increasing evidence from field and molecular studies suggests that plant responses to a combination of stresses are not simply the sum of their responses to each individual stress (Atkinson and Urwin, 2012, and references therein). Further, abiotic and biotic stresses can interact in complex ways (Suzuki *et al.*, 2014). As Blum (2016) points out, a single stress may elicit a variety of different strains. Conversely, different stresses can result in similar strains. It is time we moved from the ‘one gene–one stress’ model to a more integrative, systems-level approach.

## Defining and annotating gene function based on loss-of-function and/or overexpression studies

It was only about a decade ago that scientists working on model organisms such as mouse and Arabidopsis set out to accomplish the grand task of elucidating the function of every gene in the genome. Initially, this was to be achieved by phenotyping collections of knockout mutants generated for every gene in the genome (Alonso *et al.*, 2003; Austin *et al.*, 2004). It soon became evident that a large number of single-gene knockouts showed no discernible phenotype. There are many reasons for this phenomenon, discussed elsewhere (Bouché and Bouchez, 2001; Barbaric *et al.*, 2007); among them is the fact that the phenotype of many loss-of-function mutants is conditional; in other words, it will only be revealed under specific environmental conditions.

The identification of ‘stress resistance genes’ has frequently relied on the premise that if a phenotype is observed in a knockout mutant upon exposure to a particular stress, the function of the knocked-out gene must be associated with response to that stress. The converse is also often assumed to be true, when overexpression of a given gene leads to a measurable change in resistance to a stress. Experimental evidence from the literature of the last two decades suggests that it is not so simple. Research on plant Al resistance mechanisms provides good illustrations that certain phenotypic effects of gene knockout and overexpression are not necessarily indicative of a gene’s native function (Boxes 1 and 2).

**Box 1. Aluminum (Al) toxicity and plant Al resistance**

Aluminum toxicity is one of several components of the acid soil syndrome, which is often associated with phosphorus, nitrogen, potassium, calcium, magnesium, and micronutrient deficiencies, as well as with manganese toxicity. Furthermore, acid soils are prevalent across many environments where they occur in combination with other stresses, such as drought or short-term waterlogging. Al inhibits root growth and function, leading to nutrient and water deficiency, and resulting in greatly reduced crop yields. In the Al literature, the terms ‘tolerance’ and ‘resistance’ have frequently been applied interchangeably. For better clarity, the scientific community has recently favored use of the term ‘Al resistance’ to describe specifically the ability to sustain growth and yield on Al-toxic soils (Kochian *et al.*, 2015). Al resistance encompasses mechanisms that (i) prevent Al from entering the root (i.e. exclusion mechanisms); or (ii) detoxify or sequester Al internally (true tolerance mechanisms).

The first Al resistance genes identified encode malate [Al-activated malate transporter (ALMT)] and citrate [multidrug and toxic compound extrusion (MATE)] membrane transporters in wheat, sorghum, and barley [general model below; see also Kochian *et al.* (2015) for a comprehensive review]. Root exudation of organic acids (OAs) such as malate and citrate, which chelate Al^3+^ in the rhizosphere, is a major mechanism of plant Al resistance.

The general model shown illustrates putative Al-mediated signaling pathways (red) for perception and triggering of Al responses in roots; and mechanisms of Al resistance based on transport of OAs (blue) as chelators of Al^3+^. Al exclusion mechanisms involve the root exudation of OA anions via Al-activated anion channels (ALMTs) or carriers (MATEs) across the plasma membrane. Internal Al detoxification mechanisms involve Al^3+^ influx (dashed black arrow path) across the root cell plasma membrane into the cytosol, chelation by OA anions, followed by sequestration into the vacuole.

**Figure:**
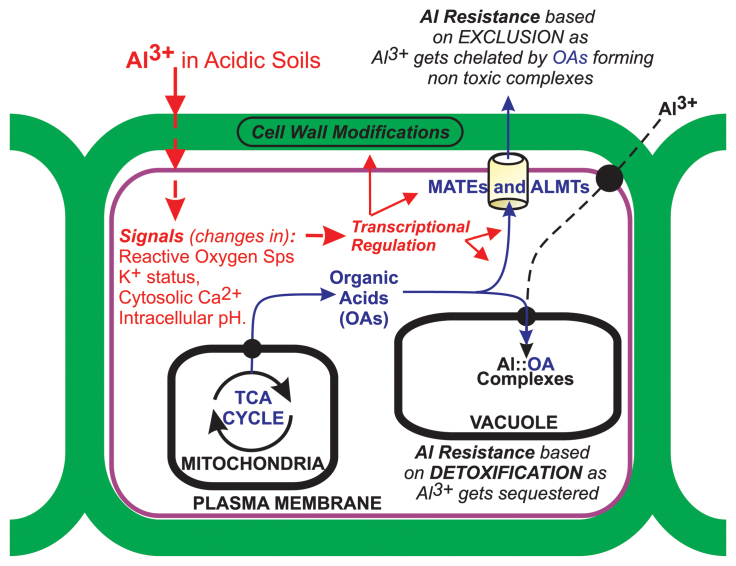

**Box 2. Lessons from plant aluminum resistance**

The cloning of wheat *TaALMT1* constituted the discovery of a novel family of plant malate-permeable anion channel transporters, named after its founding member. For historical reasons, this misleading nomenclature is still in use, even though it is now well established that many members of the ALMT family characterized to date have numerous and diverse functions beyond Al resistance. Likewise, plants harbor many MATE genes, most uncharacterized. Plant MATEs reported so far are involved in diverse physiological processes including anthocyanin accumulation, protection of roots from inhibitory compounds, iron homeostasis, and resistance to toxic metals. Plant MATE transporters related to Al resistance belong to a unique subgroup that specifically transport the organic acid (OA) anion citrate.

The function of membrane transporters is determined not only by their transport characteristics (e.g. substrate specificity), but also by their cellular and tissue-specific localization. These attributes are over-ridden when a gene is overexpressed under a constitutive promoter. Therefore, it is not surprising that overexpression of genes encoding transporters of the same family (and with similar transport characteristics), but with different functions *in planta*, can also result in increased Al resistance. For instance, *ZmALMT2* encodes an anion channel in maize that is not involved in mediating root Al exclusion, but rather plays a putative role in mineral nutrition and ion homeostasis (Ligaba *et al.*, 2012). Nevertheless, constitutive expression of *ZmALMT2* in an Al-sensitive Arabidopsis line results in an Al-independent, constitutive root malate efflux which leads to an increase in Al resistance. Likewise, the constitutive expression of a MATE citrate efflux transporter, involved in Fe–citrate chelation in the xylem for translocation to the shoots, resulted in increased Al resistance in Arabidopsis seedlings (Durrett *et al.*, 2007).

In a classic paper from the early Al literature, de la Fuente and colleagues (1997) demonstrated that the overexpression of a bacterial *citrate synthase* gene led to a 2-fold increase in Al resistance in transgenic tobacco and papaya plants. Citrate synthase is part of the Krebs cycle and necessary for the production of the OA citrate, released by roots in response to Al in certain species. Subsequently, other studies have shown similar increases in Al resistance by overexpression of genes involved in OA metabolism as well as other processes [e.g. oxidative stress and cell wall modification (see table 2 in Ryan *et al.*, 2011)]. These studies demonstrate a valid approach to increasing Al resistance by altering metabolic pathways using biotechnology. However, it would be erroneous to conclude that these are ‘Al resistance genes’. Rather, some are ‘structural’ genes (e.g. *citrate synthase*), and as such perform a basic role without which certain mechanisms of Al resistance would not be achievable; others simply provide some physiological advantage under stress. Therefore, altering their expression by overexpression indirectly affects Al resistance. Conversely, knocking out components of these pathways can lead to Al-sensitive phenotypes, not necessarily implying that the primary functions of these genes are related to Al resistance.

Particularly in the case of conditional mutants, the finding that a gene knockout is more susceptible to a particular form of stress does not exclude the possibility that it may also be susceptible to other stresses, or that it might display additional phenotypes under conditions that we would never consider testing. Take the fascinating story of an unexpected phenotype in mice: knockout mutants of the *melanocortin 5 receptor* gene (*mcr5*) appeared to have no discernible phenotype under a variety of conditions tested. However, after employing a swimming protocol generally used as a physical stress test, researchers observed that the knockout mice took longer to dry. It was demonstrated that the mutation disrupted exocrine glands, leading to decreased production and secretion of sebum (i.e. oil) (Chen *et al.*, 1997; Barbaric *et al.*, 2007).

The tale of the wet mice underscores the need for caution when assigning function to genes, and researchers should not assume that there is nothing new to discover about genes thought of as having ‘known function’. Naturally, most research programs focus their efforts on studying particular trait(s) within the realm of their scientific interest, and are unlikely to have the resources, expertise, or time to find phenotypes outside their research focus. While we are not suggesting that they should, we do suggest that their conclusions should be framed within the constraints of what they can observe in their experiments. Appropriate annotation of observed phenotypes allows the possibility that other researchers may discover new insight into the function(s) of the same genes based on different experiments.

## Standardized approaches in research on stresses will facilitate data integration

Recent advances in plant phenotyping technology (Fiorani and Schurr, 2013) have enabled the generation of vast amounts of valuable phenotypic data under a wide range of different stress conditions. The full potential of these large data sets will only be reached if they are collected, annotated, and made available to the research community using common standards, vocabularies, and protocols for recording, structuring, and querying the data (Deans *et al.*, 2015). A set of recommendations for metadata and data handling in plant phenotyping was recently developed by two large European infrastructural projects (Krajewski *et al.*, 2015). In the USA, the Planteome Project (http://www.planteome.org) is working to develop a set of common standards and reference vocabularies to describe plant biology data (including stress responses) and to standardize gene and phenotype annotation workflows.

Scientists working on abiotic stress research should encourage the development and adoption of standard methodologies for phenotyping under both controlled and field conditions to ensure the potential for data integration across experiments and platforms (Wilkinson *et al.*, 2016). This will also facilitate the timely and efficient sharing of information and materials, and help avoid unnecessary redundancy. Some form of standardization should also be encouraged for low-throughput studies, including those focused on single genes and mutants. The wide adoption of standardized methods and vocabularies will prevent many of the common mistakes found in today’s literature and is essential to a systems-level approach to the study of plant stress responses.

There is also a need to develop species-specific databases that integrate information across data domains, as they are critical to the translation of basic biological and genomics research into applications in agriculture. For example, rice is a model species with rich ‘omics’ resources. These include whole-genome sequencing/re-sequencing data sets, transcriptomes, protein–protein interactomes, metabolomes, phenomes, gene-indexed mutant populations, and the largest species-specific gene bank in the world (Chandran and Jung, 2014). These resources are scattered across different databases and, due to a lack of adherence to a set of curation and annotation standards, are currently not searchable in any co-ordinated way. Previous attempts to bring together disparate domains of information about rice have been unable to keep up with the rapid proliferation of data. The situation is especially challenging for translational researchers who wish to identify sources of stress resistance that could help accelerate plant improvement. There is an urgent need to organize and integrate the abundance of information about stress resistance from different sources to make it more accessible and relevant to applications in agriculture.

## Looking forward

Bouché and Bouchez (2001) suggested more than a decade ago that the notion of biological function refers to many layers of complexity in living organisms, and therefore can only be defined using a variety of experimental methods. Thanks to the many technological advances of the past decade, the researchers of today can make this a reality. We have the opportunity to look at plant responses to stress at a genome-wide scale using an array of techniques, and to integrate multiple layers of information into systems that support complex user querying at a systems level. However, to achieve this goal requires that the plant research community organize itself around a set of standardized experimental and data annotation protocols so that data integration and sharing is possible. Enabling researchers to access and share information more easily has the potential to accelerate greatly the pace of discovery in plant research. It also means that research can become better integrated with the efforts of the plant breeding community to accelerate the development of crop plants that are able to maintain high yields in the face of fluctuating and stressful environmental conditions.
